# Study of the Structural and Magnetic Properties of Co-Substituted Ba_2_Mg_2_Fe_12_O_22_ Hexaferrites Synthesized by Sonochemical Co-Precipitation

**DOI:** 10.3390/ma12091414

**Published:** 2019-04-30

**Authors:** Tatyana Koutzarova, Svetoslav Kolev, Kiril Krezhov, Borislava Georgieva, Daniela Kovacheva, Chavdar Ghelev, Benedicte Vertruyen, Frederic Boschini, Abdelfattah Mahmoud, Lan Maria Tran, Andrzej Zaleski

**Affiliations:** 1 Institute of Electronics, Bulgarian Academy of Sciences, 72 Tsarigradsko Chaussee, 1784 Sofia, Bulgaria; svet_kolev@yahoo.com (S.K.); kiril.krezhov@gmail.com (K.K.); b.georgiewa@abv.bg (B.G.); chavdarghelev@yahoo.com (C.G.); 2 Institute of General and Inorganic Chemistry, Bulgarian Academy of Sciences, Acad. Georgi Bonchev Str., bld. 11, 1113 Sofia, Bulgaria; dkovacheva@gmail.com; 3 Greenmat, Chemistry Department, University of Liege, 11 allée du 6 août, 4000 Liège, Belgium; b.vertruyen@uliege.be (B.V.); frederic.boschini@uliege.be (F.B.); abdelfattah.mahmoud@uliege.be (A.M.); 4 Institute of Low Temperature and Structure Research, Polish Academy of Sciences, ul. Okólna 2, 50-422 Wroclaw, Poland; l.m.tran@intibs.pl (L.M.T.); a.zaleski@intibs.pl (A.Z.)

**Keywords:** sonochemical co-precipitation, Y-type hexaferrite, Mossbauer spectroscopy, magnetic properties, magnetic-phase transition

## Abstract

Ba_2_Mg_0.4_Co_1.6_Fe_12_O_22_ was prepared in powder form by sonochemical co-precipitation and examined by X-ray diffraction, Mössbauer spectroscopy and magnetization measurements. Careful XRD data analyses revealed the Y-type hexaferrite structure as an almost pure phase with a very small amount of CoFe_2_O_4_ as an impurity phase (about 1.4%). No substantial changes were observed in the unit cell parameters of Ba_2_Mg_0.4_Co_1.6_Fe_12_O_22_ in comparison with the unsubstituted compound. The Mössbauer parameters for Ba_2_Mg_0.4_Co_1.6_Fe_12_O_22_ were close to those previously found (within the limits of uncertainty) for undoped Ba_2_Mg_2_Fe_12_O_22_. Isomer shifts (0.27–0.38 mm/s) typical for high-spin Fe^3+^ in various environments were evaluated and no ferrous Fe^2+^ form was observed. However, despite the indicated lack of changes in the iron oxidation state, the cationic substitution resulted in a significant increase in the magnetization and in a modification of the thermomagnetic curves. The magnetization values at 50 kOe were 34.5 emu/g at 4.2 K and 30.5 emu/g at 300 K. The zero-field-cooled (ZFC) and field-cooled (FC) magnetization curves were measured in magnetic fields of 50 Oe, 100 Oe, 500 Oe and 1000 Oe, and revealed the presence of two magnetic phase transitions. Both transitions are shifted to higher temperatures compared to the undoped compound, while the ferrimagnetic arrangement at room temperature is transformed to a helical spin order at about 195 K, which is considered to be a prerequisite for the material to exhibit multiferroic properties.

## 1. Introduction

Multiferroic materials form a special category of magnetic materials characterized by the coexistence of long-range magnetic and ferroelectric orders, a property that has provoked keen interest from both fundamental and applied points of view [1,2,3,4]. In particular, magnetoelectric multiferroics combine coupled electric and magnetic dipoles [5,6]. Recent research has demonstrated that the magnetoelectric effect can be induced by complex internal arrangements of magnetic moments in some hexaferrites [5,6,7,8,9,10]. One of the first magnetoelectric hexaferrites discovered was Ba_2_Mg_2_Fe_12_O_22_. It possesses a somewhat relatively high spiral-magnetic transition temperature (~200 K), exhibits multiferroic properties at zero magnetic field, while one can manipulate the direction of the electric polarization using relatively weak magnetic fields (<0.02 T) [11] compared with the other hexaferrites [5,12]. In this material, the magnetically induced electrical polarization arises from noncollinear spiral magnetic orders [5] and is described well by the spin-current model [13,14].

Ba_2_Mg_2_Fe_12_O_22_ is representative of the family of Y-type hexaferrites, Ba_2_Me_2_Fe_12_O_22_, (Me being a divalent cation) with a structure belonging to the space group R-3m. All cations (Me^2+^ and Fe^3+^) are positioned in six specific crystallographic sites: two tetrahedral sites (6c_IV_ and 6c∗_IV_) and four octahedral sites (3a_VI_, 3b_VI_, 6c_VI_, and 18h_VI_). The divalent cations’ species and the site they occupy in the lattice cell may bring significant modifications of the structural and magnetic parameters. The unit cell involves three formula units and consists of consecutively stacked S (spinel Me_2_Fe_4_O_8_) and T (Ba_2_Fe_8_O_14_)-blocks along the hexagonal *c* axis in a sequence of (TST’ST”S”), with the primes indicating rotation about the c-axis by 120 degrees [15]. The S-blocks comprise two spinel units of two layers of four oxygen atoms with three metal atoms between each layer, in four octahedral sites with the cation being enclosed by six oxygen anions and two tetrahedral sites where four oxygen anions encircle the cation [15]. The T-block is made of four oxygen layers, with a barium atom substituting an oxygen atom in the inner two layers, which are opposite one another in the neighboring layers, resulting in two tetrahedral and six octahedral sites [15]. The easy magnetization axis lies in a plane perpendicular to the *c* axis direction, while the non-compensated magnetic moment lying in the *ab* plane arises from dominating majority spins in octahedral 3*a*, 3*b*, and 18*h* sites and minority spins in tetrahedral 6*c_T_* and 6*c_S_* and octahedral 6*c* sites [16]. These majority spins and minority spins determine the two magnetic sublattices different from crystal structural blocks—*L_m_* (spins in octahedral 3*a*, 3*b*, and 18*h* sites) and *S_m_* (spins in tetrahedral 6*c_T_* and 6*c_S_* and octahedral 6*c* sites) blocks alternating along [001], which bear, correspondingly, opposite large and small magnetization *M* [11]. For Ba_2_Mg_2_Fe_12_O_22_ at temperatures exceeding room temperature (below 553 K), a ferrimagnetic spin arrangement sets in, and below 195 K a proper screw spin structure (helical) was identified [17,18]. The turn angle of the helix is about 70° [13]. 

Magnetic measurements of Ba_2_Mg_2_Fe_12_O_22_ single crystals have demonstrated a spin reorientation transition to a longitudinal-conical spin state below about 50 K, enabling one to control the electric polarization by a weak magnetic field [19]. 

These two magnetic phase transitions are the main reasons for observing the magnetoelectric effect in Ba_2_Mg_2_Fe_12_O_22_. The application of hexaferrites as magnetoelectric materials prompts a continuous demand for ways to increase the temperature of these transitions, two commonly adopted paths being the substitution of magnetic cations with non-magnetic and, inversely, of non-magnetic cations with magnetic ones.

Thus, it was reported that the Co^2+^ substitution in M-type and W-type hexaferrites raises the magnetic saturation and the coercivity [20,21]. In addition, the Co substitution can enhance the magnetoelectric effect in hexaferrites, as established by Beevers et al. [20]. 

Wu et al. studied the dielectric properties and *ac* conductivity of cobalt-substituted Ba_2_Mg_2−*x*_Co_*x*_Fe_12_O_22_ (*x* = 0.4, 0.8, 1.2, 1.6). They investigated in particular the influence of Co^2+^ substitution of Mg^2+^ on the magnetic properties and the magnetic phase transition at a magnetic field of 500 Oe. They found that the transition temperature from a ferromagnetic to a helical spin arrangement increases with increasing the cobalt concentration, and for *x* = 1.6 it is 226 K. In the case of Ba_2_Co_2_Fe_12_O_22_ (fully substituted), this magnetic phase transition occurs at 225 K [22] and the *M_s_* and *H*_c_ are 32 emu/g and 70 Oe, respectively [23,24]. The Co substitution also increases the Curie temperature from 553 K for Ba_2_Mg_2_Fe_12_O_22_ to 613 K for Ba_2_Co_2_Fe_12_O_22_ [17,18,22].

In our previous studies [25,26], we showed that in a Ba_2_Mg_2_Fe_12_O_22_ powder material synthesized by sonochemical co-precipitation, the magnetic phase transition from ferromagnetic-to-spiral spin order occurs at 196 K at a magnetic field of 100 Oe.

In some of our earlier works, we have studied in detail the effect of the preparation technique on the microstructural and magnetic properties of Ba_2_Mg_2_Fe_12_O_22_ powders [25,26]. We found that using sonochemical co-precipitation results in the formation of Ba_2_Mg_2_Fe_12_O_22_ particles with perfect hexagonal shape, as opposed to the case of performing auto-combustion; moreover, better magnetic properties were achieved. Similar results were reached for other types of Y-type hexaferrites [27]. In brief, it can be thought of as a modified co-precipitation, where the formation of the precursor particles occurs in a liquid when acted upon by a high-power ultrasound wave. This causes the formation, growth and implosive collapse of bubbles with very short life-times that are produced in hot spots with effective temperatures of about 5000 K, high pressures (800 atm) and heating and cooling rates above 1010 K s^−1^ [28,29,30].

This paper reports a study on the effect of cobalt substitution for magnesium in the Y-type Ba_2_Mg_2_Fe_12_O_22_ hexaferrite on its structure and magnetic characteristics. We pay special attention to the changes in the magnetic-phase transition temperature due to the partial major substitution of the nonmagnetic Mg^2+^cations with magnetic Co^2+^ cations and to the influence of an external magnetic field. The Co^2+^ was chosen because it has a similar ionic radius (0.58Å) as Mg^2+^ (0.57Å) [31], which enables us to investigate the effect of another transition element on the magnetic characteristics of Y-type hexagonal Ba-ferrite.

## 2. Materials and Methods

The Ba_2_Mg_0.4_Co_1.6_Fe_12_O_22_ powder material was synthesized by sonochemical co-precipitation. Stoichiometric amounts of corresponding metal nitrates, such as Co(NO_3_)_2_·6H_2_O, Mg(NO_3_)_2_·6H_2_O and Fe(NO_3_)_3_·9H_2_O, and 10 wt% of over-stoichiometric Ba(NO_3_)_2_, were dissolved completely in deionized water and homogenized; the co-precipitation process was triggered by adding NaOH at pH = 11.5. The process was intensified via high-power ultrasound stirring enhancing the reaction rate, the mass transport and the heat effects. The ultrasound with amplitude 40% was applied for 15 min by a Sonics ultrasonic processor (750 W). The precipitate was separated in a centrifuge, dried and milled. The as-obtained precursor was calcined at 700 °С in air and then subjected to heat treatment at 1170 °С for seven hours in air to obtain the final material—Ba_2_Mg_0.4_Co_1.6_Fe_12_O_22_.

The as prepared Ba_2_Mg_0.4_Co_1.6_Fe_12_O_22_ powders were characterized by X-ray diffraction (XRD, Bruker AXS GmbH, Karlsruhe, Germany) for phase identification, Mӧssbauer spectroscopy (MS, Wissenschaftliche Elektronik GmbH, Ortenberg, Germany) and magnetization measurements. The XRD patterns were taken at ambient temperature on a Brucker D8 diffractometer (40 kV, 30 mA, Bruker AXS GmbH, Karlsruhe, Germany) controlled by a DIFFRAC*plus*BASIC software (Version 2.6.1), in Bragg-Brentano reflection geometry with Cu-Kα radiation (λ = 1.5418 Å) and LynxEye detector. Rietveld quantification analysis was performed using Topas 4.2 (Bruker AXS GmbH, Karlsruhe, Germany).

Iron-57 Mössbauer spectroscopy (Wissenschaftliche Elektronik GmbH, Ortenberg, Germany) was used to probe the oxidation state of the iron ions and investigate their coordination. The ^57^Fe transmission MS data were registered by a constant-acceleration spectrometer with a ^57^Co(Rh) source at room temperature. The Mössbauer spectrometer is equipped with Wissel velocity drive using a compatible CMCA-2000 multichannel analyzer Wissel unit for data acquisition (Wissenschaftliche Elektronik GmbH, Ortenberg, Germany). The Mӧssbauer spectrum absorber was prepared using 40 mg/cm^2^ of material mixed with boron nitride. The spectrometer was calibrated at room temperature with the magnetically split sextet spectrum of a high-purity α-Fe foil as a reference absorber. The measurements were conducted in the (±12 mm/s) velocity range. The spectral parameters, namely, the isomer shift (δ), the quadrupole splitting (Δ), the linewidth (Γ), the magnetic field (*B*_hf_) and the relative resonance areas of the different spectral components were determined by fitting the experimental data. The validity of the fit was estimated based on minimizing the number of parameters and χ^2^ values. 

A PPMS equipped with an ACMS option (Quantum Design Inc., San Diego, CA, USA) was used to follow the material’s magnetic characteristics. The hysteresis loops were plotted at 4.2 K and at room temperature. The magnetization dependence on the temperature was followed in zero-field-cooled (ZFC) and field-cooled (FC) protocols in magnetic fields of 50 Oe, 100 Oe, 500 Oe and 1 kOe. In the ZFC procedure, the sample under study was cooled from room temperature to 4.2 K without any magnetic field, and the magnetization was registered during heating from 4.2−300 K at a heating rate of 3 K/min in the respective applied magnetic field. The FC curve was measured on the same sample upon cooling from 300 K to 4.2 K under the same magnetic field.

## 3. Results and Discussion

**Figure 1:**
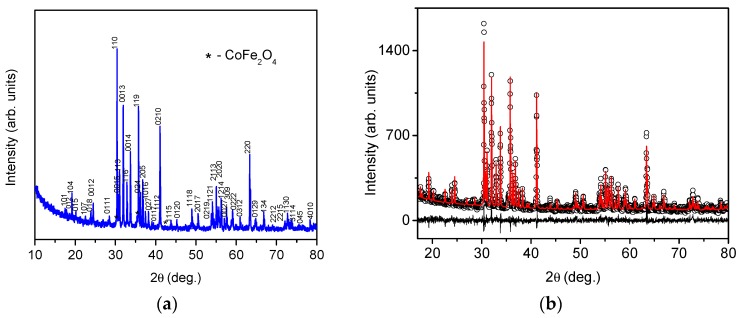
XRD pattern of the Ba_2_Mg_0.4_Co_1.6_Fe_12_O_22_ powder (**a**) and Rietveld plot for Ba_2_Mg_0.4_Co_1.6_Fe_12_O_22_ powder (black dots—experimental, red line—calculated, black line—difference) (**b**).

The preliminary analysis of the diffraction pattern of the Ba_2_Mg_0.4_Co_1.6_Fe_12_O_22_ showed that, besides the peaks corresponding to the Y-type hexaferrite phase, traces of a second phase with a spinel type structure were present. The Rietveld plot of the Ba_2_Mg_0.4_Co_1.6_Fe_12_O_22_ powder material is shown in Figure 1. The well-defined sharp Bragg peaks indicate a good crystalline quality of the hexaferrite phase. As starting models for the Rietveld refinement, the structure of BaCoFe_6_O_11_ (file 1008434 in the Crystallography Open Database) and the structure of CoFe_2_O_4_ (file 1533163 in the Crystallography Open Database) were taken. The reliability factors for the fit are as follows: R_exp_ = 8.26, R_wp_ = 9.18, R_p_ = 7.22, GOF = 1.11, DW = 1.65, Y-type hexaferrite R-Bragg = 3.33, CoFe_2_O_4_ R-Bragg = 1.07. The Rietveld quantification results showed that the quantity of the spinel phase was less than 2%. In fact, the mass content of this spinel impurity phase was evaluated to be 1.4 ± 0.6%. This small amount of a second phase did not strongly affect the sample’s magnetic properties, as can be seen in Figure 3.

The unit cell parameters and cell volume of the Y-hexaferrite phase were variable parameters during the Rietveld quantification and were refined by the program. The lattice constant *a* and *c* are 5.8613(1) Å and 43.503(1) Å, respectively. These results for unit cell parameters are in good agreement with the values published earlier for the Ba_2_Mg_2_Fe_12_O_22_ and Ba_2_Co_2_Fe_12_O_22_ hexaferrites [17,19,32,33,34]. As expected, despite the significant degree of substitution, no drastic changes were observed in the unit cell parameters of Ba_2_Mg_0.4_Co_1.6_Fe_12_O_22_ due to the similar ionic radii of Co^2+^ (0.58 Å) and Mg^2+^ (0.57 Å) [31] cations.

**Figure 2:**
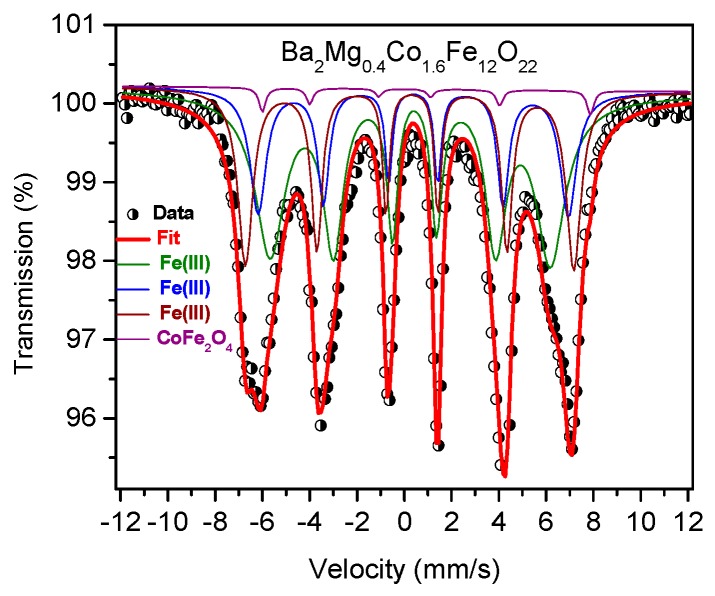
The Mössbauer spectrum of Ba_2_Mg_0.4_Co_1.6_Fe_12_O_22_ material recorded at room temperature. The black dots and red solid lines refer to the experimental data and to the fit of the spectrum, respectively.

The results of determining the iron ions’ state of oxidation and their coordination in Ba_2_Mg_0.4_Co_1.6_Fe_12_O_22_ powder by means of ^57^Fe MS at room temperature are presented in Figure 2. Table 1 summarizes the respective hyperfine parameters. The Mössbauer spectrum revealed the presence of magnetic ordering at room temperature manifested as relatively well-resolved sextets, which makes it possible to arrive at a rather unique fitting model; no doublet or singlet related to superparamagnetic particles or paramagnetic phases were observed.

The spectrum was simulated with three magnetic components corresponding to the three different types of coordination of the ferromagnetic Fe atoms (tetrahedral in S and T blocks, octahedral in S and T block and octahedral in T-S block) in the structure of Ba_2_Mg_0.4_Co_1.6_Fe_12_O_22_ and one sextet corresponding to the CoFe_2_O_4_ as a minor secondary phase (<2%). This result confirmed that the material was magnetically ordered at room temperature. The Mӧssbauer spectral shape of Ba_2_Mg_0.4_Co_1.6_Fe_12_O_22_ and Ba_2_Mg_0.5_Co_1.5_Fe_12_O_22_ are very similar suggesting that no changes occurred in the iron oxidation state and local environment of iron as the Co content was raised [35]. A good-quality fit of the Mössbauer spectrum of Ba_2_Mg_0.4_Co_1.6_Fe_12_O_22_ was obtained by using only four doublets attributed to three Fe^3+^ components in Ba_2_Mg_0.4_Co_1.6_Fe_12_O_22_ [36], which is in good agreement with the Mössbauer results of the non-substituted Y-type hexaferrite Ba_2_Mg_2_Fe_12_O_22_, in which three different electric field gradient (EFG) axes with respect to the spin direction [110] have been shown [18]. Indeed, the Mössbauer spectrum of Ba_2_Mg_0.4_Co_1.6_Fe_12_O_22_ is a superposition of three sub-spectra originating from the six different iron positions as revealed by Nakamura et al. [18]. The fourth average sextet corresponds to the two Fe^3+^ sites in CoFe_2_O_4_ [37]. The Mössbauer parameters obtained for Ba_2_Mg_0.4_Co_1.6_Fe_12_O_22_ are close to those previously found (within the limits of uncertainty) for Ba_2_Mg_2_Fe_12_O_22_, which confirms that the substitution of Mg by Co did not substantially affect the oxidation state and the local environment of iron in Ba_2_Mg_0.4_Co_1.6_Fe_12_O_22_. The isomer shift values (0.27–0.38 mm/s) are typical of high-spin Fe^3+^ in different environments and no ferrous Fe^2+^ form was observed. The quadrupole splitting values (−0.18–0.0 mm/s) confirm the presence of different iron environments in the substituted Ba_2_Mg_0.4_Co_1.6_Fe_12_O_22_ that reflect three different kinds of local coordination. The asymmetry of different iron sites is affected by the partial substitution of Co by Mg. Indeed, the quadrupole splitting values in Ba_2_Mg_0.4_Co_1.6_Fe_12_O_22_ are slightly higher than those in undoped Ba_2_Mg_2_Fe_12_O_22_ (−0.10–0.00 mm/s). The hyperfine magnetic fields of the three magnetic components are different, 37.1 T, 40.7 T and 43.1 T, which is in agreement with the previous studies [18,36]. The occupancy of Fe ions at the three crystallographic positions in the structure can be obtained from the relative area of each sextet in the corresponding Mӧssbauer spectrum by assuming similar Mӧssbauer Lamb factors for all sites. The three sub-spectral components show different relative intensities of 50%, 21%, and 27%, in good agreement with the results quoted in the literature.

**Figure 3:**
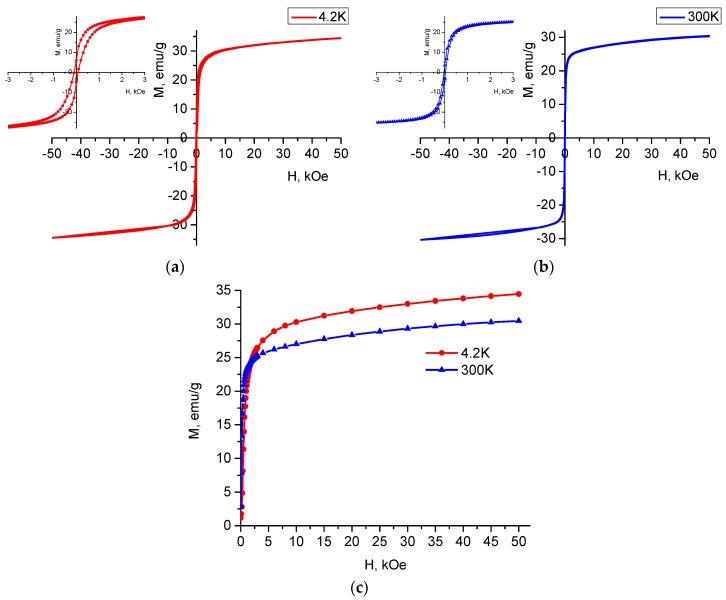
Hysteresis curves (**a**) at 4.2 K and (**b**) 300 K. The insets in (**a**,**b**) show expanded view of the narrow magnetic hysteresis loops up to 3 kOe; (**c**) shows the initial magnetization curves at 4.2 K and 300 K.

The hysteresis loops of the powder material at room temperature and 4.2 K are displayed in Figure 3 and provide evidence that the amount of cobalt ferrite did not appreciably affect the basic magnetic properties of the material. The magnetic parameters, namely, the magnetization at 50 kOe (*M* (50 kOe)) and the coercive field (*H*_c_) obtained from the curves are listed in Table 2. The coercive field was determined from the hysteresis curve as half of the difference of the magnetic field value at zero magnetization and the *M* (50 kOe) was determined from the initial magnetization curves.

Saturation of the initial magnetization curves is seen (Figure 3c) at a magnetic field of 50 kOe, the magnetization values being 34.5 emu/g and 30.4 emu/g at 4.2 K and 300 K, respectively. The *M* (50 kOe) values for 300 K are close to those reported by Wu et al. [38] and lie between the values reported for non-substituted Ba_2_Mg_2_Fe_12_O_22_ (23 emu/g) and Ba_2_Co_2_Fe_12_O_22_ (34 emu/g) [13,23]. The hysteresis curve at 300 K is very narrow (about 70 Oe), while *H*_c_ is about 35 Oe. This value is typical for hexaferrites whose magneto-crystalline anisotropy is planar. This *H*_c_ value is slightly higher than that for unsubstituted Ba_2_Mg_2_Fe_12_O_22_ (12.21 Oe) prepared following a similar route [26], but is lower than that observed for the Ba_2_Co_2_Fe_12_O_22_ (70 Oe) synthesized by a solid-state method [23]. The *M* (50 kOe) value at 4.2 K is not much different than that for unsubstituted Ba_2_Mg_2_Fe_12_O_22_ (33.57 emu/g) obtained under similar conditions [26]. The *M_r_* values for the two temperatures 4.2 K and 300 K are very low, which yields also a very low squareness ratio (*M_r_/M_s_*), indicating a multi-domain structure of the particles in the powder.

It is well known that CoFe_2_O_4_ is a hard magnetic material with high coercivity and moderate magnetization [39]. Apparently, the very small amount of the impurity phase in our material prevented possible changes in the hysteresis curves to be revealed that could support the presence of a hard and/or soft magnetic material.

Figure 3c illustrates that the magnetization curves at 4.2 K and 300 K recorded in fields up to 50 kOe do not saturate. In view of studying in detail the behavior of the substituted magnetic compound at low temperatures, sets of thermomagnetic curves were registered in various magnetic fields up to 1 kOe in both zero-field-cooled (ZFC) and field-cooled (FC) modes.

**Figure 4:**
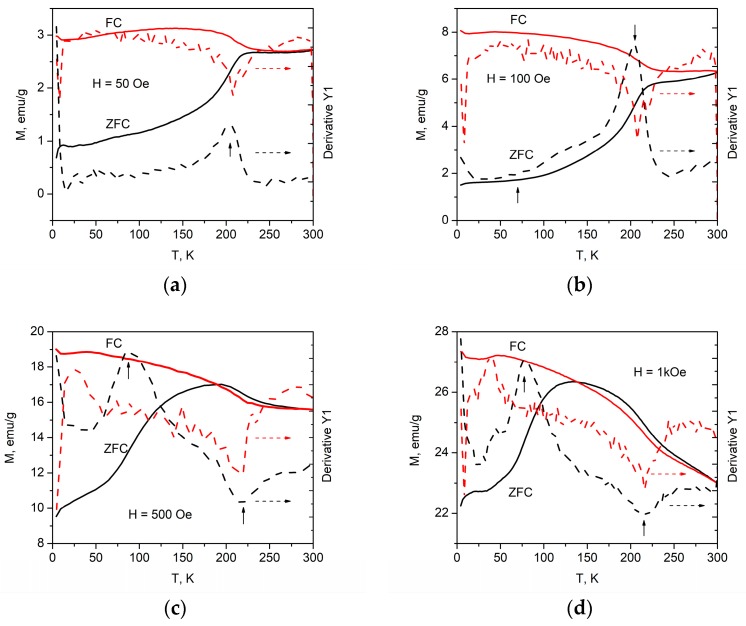
Temperature dependence of zero-field-cooled (ZFC) and field-cooled (FC) magnetization at a magnetic field of (**a**) 50 Oe, (**b**) 100 Oe, (**c**) 500 Oe and (**d**) 1 kOe.

The magnetic-phase transition temperature can be derived from the magnetization’s temperature dependence at a fixed applied magnetic field. Figure 4 presents the ZFC and FC magnetization curves of the Ba_2_Mg_0.4_Co_1.6_Fe_12_O_22_ powder as functions of the temperature in magnetic fields of 50 Oe, 100 Oe, 500 Oe and 1 kOe in the temperature range 4.2–300 K, wherein the dashed lines correspond to the plot of d*M*/d*T* vs. *T*. The transition temperature determined from the first derivative of the ZFC and FC magnetization curves is indicated by an arrow. 

As can be seen in Figure 4a, as the temperature is increased above 4.2 K, the ZFC magnetization rises very rapidly up to 12 K. This finding is only clearly pronounced in a field of 50 Oe (see Figure 4a) and is obscured at higher magnetic fields (see Figure 4b). Then it rises slowly up to 115 K followed by a more rapid increase between 116 K and 225 K. The magnetization reaches its maximum at 236 K and remains practically constant with the further rise of the temperature. At low temperatures, the ZFC magnetization in a field of 100 Oe slowly increases with the rising temperature and then rises rapidly between 120 K and 215 K; this is followed by a slow increase (Figure 4b). This temperature connected to the helimagnetic phase transition is higher than that observed by Behera et al. [22] for the material obtained by a classical solid-state reaction and similar to the one of Ba_2_Co_2_Fe_12_O_22_ observed by the same authors. This shows that the synthesis conditions also influence the magnetic-phase transition temperature. The dependence of the ZFC magnetization on the temperature in a field of 500 Oe has a somewhat different behavior than those for fields of 50 Oe and 100 Oe. It rises rapidly up to 180 K, reaches its maximum at 191 K and then decreases. As seen in Figure 4c, a superposition of the ZFC and FC curves takes place at 285 K. Figure 4d shows that although the ZFC magnetization dependence on the temperature for the field of 1 kOe has a similar behavior as the curve at 500 Oe, it reaches a maximum at the much lower temperature of 135 K.

The first derivatives of the FC magnetization vs. temperature curves show minima at 207 K, 208 K, 220 K and 218 K for magnetic fields of 50 Oe, 100 Oe, 500 Oe and 1 kOe, respectively. These minima correspond to inflection points of the *M*_FC_ vs. *T* curve. The inflection points of the ZFC magnetization curves indicating changes in the magnetic arrangement were observed at similar temperatures.

The members of the Ba_2_Mg_2−*x*_Co_*x*_Fe_12_O_22_ system, including the undoped compound (*x* = 0.0), are paraelectric in the absence of an applied magnetic field. It was reported in [19] for the case of Ba_2_Mg_2_Fe_12_O_22_ single crystals that when the temperature drops below room temperature in zero magnetic field, the magnetic arrangement is transformed at around 195 K from a collinear ferrimagnetic with a ferromagnetic moment in the (001) plane to a proper screw spin ordering. A second transition to a longitudinal-conical spin arrangement takes place at around 50 K, whose ferromagnetic moment provides the possibility for controlling the electrical polarization in a low magnetic field applied along (001).

For the substitution degree *x* = 1.6, we observed that the first magnetic structure change was shifted to the higher temperature range. An increase of this transition temperature, as well as the saturation magnetization *M_s_*, by increasing the Co content was reported by Wu et al [38] in their study on Ba_2_Mg_2−*x*_Co_*x*_Fe_12_O_22_ (*x* = 0.4, 0.8, 1.2, 1.6); but as shown above, we observed in addition that the shift was rather sensitive to the applied magnetic field. This should be related to the magnetic anisotropy which is both temperature and magnetic-field dependent. As expected, the magnetic anisotropy seems to affect the ZFC curve more strongly. Indeed, from Figure 9 in [38], one may extract roughly the same value of 220 K for the transition temperature as we determined from both the FC and ZFC magnetization curves.

For the second transition temperature, we determined 85 K, which was in good agreement with the value obtained at 500 Oe by Wu et al. [38] for *x* = 1.6. However, for 1 kOe the magnetic transition temperature decreased to 78 K. The reason for this decrease is still unknown. Nevertheless, such a shift in the magnetic transition temperature above the liquid nitrogen temperature suggests future studies using structure-sensitive methods, including neutron diffraction. 

The inflection point observed in the ZFC-FC magnetization curves is related to a magnetic phase transition from a ferrimagnetic to a helical spin order. Such a transition is responsible for the multiferroic properties of this material. A similar behavior was reported for Ba_2_Co_2−*x*_Zn_*x*_Fe_12_O_22_ [40]. 

## 4. Conclusions

Ba_2_Mg_0.4_Co_1.6_Fe_12_O_22_ powder material was prepared by the sonochemical co-precipitation route. The substantial cobalt substitution for magnesium in the Ba_2_Mg_2_Fe_12_O_22_ basic composition did not lead to a significant change in the unit cell parameters, but the magnetization increased substantially. The oxidation state of the iron ions and their coordination were established by iron-57 Mössbauer spectroscopy at room temperature. The isomer shift values (0.27–0.38 mm/s) are consistent with high spin Fe^3+^ in different environments; no ferrous Fe^2+^ form was observed. The quadrupole splitting values (−0.18–0.0 mm/s) confirm the presence of different iron environments in the substituted Ba_2_Mg_0.4_Co_1.6_Fe_12_O_22_, which suggests three different kinds of local coordination. The lack of changes in the iron oxidation state supports earlier evidence for preferred site occupation of magnesium and cobalt cations implying that the partial replacement of Mg^2+^ by Co^2+^ takes place in the octahedral sites. The ZFC-FC magnetization curves in different magnetic fields (50 Oe, 100 Oe, 500 Oe, 1 kOe) reveal the occurrence of two magnetic phase transitions that both shifted to higher temperatures in comparison with the undoped compound; these shifts depend on the applied magnetic field. Thus, the Co^2+^ substitution for magnesium in the Y-type Ba_2_Mg_2−*x*_Co_*x*_Fe_12_O_22_ hexaferrite family results in increasing the temperature of the setting in spin arrangements, which for the end materials (*x* = 0 and *x* = 2) are observed as the specific helical structures that are believed to be a precondition for their multiferroic behavior. Such a shift in the magnetic transition temperature above the liquid nitrogen temperature might facilitate further studies by structure sensitive methods, such as neutron diffraction, combined with other advanced techniques proving multiferroicity.

## Figures and Tables

**Table 1:** Hyperfine parameters^1^ of the room-temperature Mössbauer spectrum of Ba_2_Mg_0.4_Co_1.6_Fe_12_O_22_.

Iron Sites	δ (mm s^−1^)	Δ (mm s^−1^)	Γ (mm s^−1^)	B_hf_ (T)	Area (%)
a-Fe(III)	0.34 (1)	−0.18 (1)	0.40 (1)	37.1 (1)	50 (1)
b-Fe(III)	0.38 (2)	0.00	0.28 (2)	40.7 (1)	21 (1)
c-Fe(III)	0.27 (1)	−0.09 (1)	0.28 (1)	43.1 (1)	27 (1)
CoFe_2_O_4_	0.48 (2)	0.92 (2)	0.33 (1)	43.0 (1)	2 (1)

^1^ δ—Isomer shift, referred to α-iron at 295 K; Δ—quadrupole splitting, Γ—linewidth, Bhf—hyperfine field.

**Table 2:** Magnetic properties of Ba_2_Mg_0.4_Co_1.6_Fe_12_O_22_.

Sample	*T* K	*M* (50 kOe) emu/g	*H*_c_ Oe
Ba_2_Mg_0.4_Co_1.6_Fe_12_O_22_	300	30.4	35
Ba_2_Mg_0.4_Co_1.6_Fe_12_O_22_	4.2	34.5	95
